# The impact of HIV and ART exposure during pregnancy on fetal growth: a prospective study in a South African cohort

**DOI:** 10.1186/s12884-023-05743-x

**Published:** 2023-06-03

**Authors:** Asanda Mtintsilana, Shane A. Norris, Siphiwe N. Dlamini, Lukhanyo H. Nyati, David M. Aronoff, John R. Koethe, Jeffrey A. Goldstein, Alessandra Prioreschi

**Affiliations:** 1 SA MRC/Wits Developmental Pathways for Health Research Unit (DPHRU), Department of Paediatrics, Faculty of Health Sciences, School of Clinical Medicine, University of the Witwatersrand, Private Bag X3, Johannesburg, 2050 South Africa; 2 DSI-NRF Centre of Excellence in Human Development, University of the Witwatersrand, Johannesburg, Gauteng South Africa; 3 School of Human Development and Health, University of Southampton, Southampton, UK; 4 School of Physiology, Faculty of Health Sciences, University of the Witwatersrand, Johannesburg, South Africa; 5 Faculty of Community and Health Sciences, University of the Western Cape, Blanckenberg Street, Bellville, Cape Town 7535 South Africa; 6 Department of Medicine, Indiana University School of Medicine, Indianapolis, IN 46202 USA; 7 Division of Infectious Diseases, Department of Medicine, Vanderbilt University Medical Center, Nashville, TN 37232 USA; 8 Vanderbilt Institute for Global Health, Vanderbilt University Medical Center, Nashville, TN 37232 USA; 9 Department of Pathology, Feinberg School of Medicine, Northwestern University, Chicago, IL USA

**Keywords:** Fetal growth, HIV exposure, ART, Placental morphology, Sex differences, Fetus

## Abstract

**Background:**

*In utero* exposure to human immunodeficiency virus (HIV) and antiretroviral (ART) is associated with adverse birth outcomes, which are often attributed to alterations in placental morphology. This study used structural equation models (SEMs) to examine the impact of HIV and ART exposure on fetal growth outcomes and whether these associations are mediated by placental morphology in urban-dwelling Black South African women.

**Methods:**

This prospective cohort study included pregnant women living with HIV (WLWH, n = 122) and not living with HIV (WNLWH, n = 250) that underwent repeated ultrasonography during pregnancy, and at delivery, to determine fetal growth parameters in Soweto, South Africa. The size and the velocity of fetal growth measures (i.e., head and abdominal circumference, biparietal diameter, and femur length) were calculated using the Superimposition by Translation and Rotation. Placenta digital photographs taken at delivery were used to estimate morphometric parameters and trimmed placental weight was measured. All WLWH were receiving ART for the prevention of vertical transmission of HIV.

**Results:**

A trend towards a lower placental weight and significantly shorter umbilical cord length was reported in WLWH compared to their counterparts. After sex stratification, umbilical cord length was significantly shorter in males born to WLWH than in male fetuses born to WNLWH (27.3 (21.6–32.8) vs. 31.4 (25.0–37.0) cm, p = 0.015). In contrast, female fetuses born to WLWH had lower placental weight, birth weight (2.9 (2.3–3.1) vs. 3.0 (2.7–3.2) kg), and head circumference (33 (32–34) vs. 34 (33–35) cm) than their counterparts (all p ≤ 0.05). The SEM models showed an inverse association between HIV and head circumference size and velocity in female fetuses. In contrast, HIV and ART exposure was positively associated with femur length growth (both size and velocity) and abdominal circumference velocity in male fetuses. None of these associations appeared to be mediated via placental morphology.

**Conclusion:**

Our findings suggest that HIV and ART exposure directly affects head circumference growth in females and abdominal circumference velocity in male fetuses; but may improve femur length growth in male fetuses only.

**Supplementary Information:**

The online version contains supplementary material available at 10.1186/s12884-023-05743-x.

## Introduction

Human immunodeficiency virus (HIV) prevalence remains high in Eastern and Southern Africa [1]. This region continues to be disproportionately more affected than other regions, accounting for 54% (20.6 million) of all people living with HIV in the world in 2021 [1]. In contrast, only 2.3 million people were living with HIV in Western and Central Europe, and North America [1]. Regrettably, women and girls remain the most vulnerable group to HIV infection. In 2021, about 24.5% of South African females (aged 15 to 49 years) were living with HIV [1]. Satisfactorily, over 96% of women who were pregnant and living with HIV in South Africa (SA) had access to antiretroviral therapy (ART) to prevent mother-to-child transmission (PMTCT) in 2021, resulting in a vertical transmission rate of 3.5% [1]. Despitethe benefits of early initiation of ART before or during pregnancy, several HIV and ART-exposed but uninfected infants continue to be at risk of adverse birth outcomes, including low birth weight (LBW) and length, reduced head circumference, microcephaly, preterm birth (PTB), and small-for-gestational age (SGA) birth [2–5]. These outcomes are associated with long-term health consequences, including the risk of developing metabolic disorders, cardiovascular diseases, and neurological disorders across the life course [3, 6, 7]. Therefore, there is an urgent need for prospective studies to delineate the effect of HIV and its treatment on fetal growth outcomes.

Most previous studies investigating the effects of HIV and ART on development are based on proxy measures of growth outcomes (e.g., birth weight) and often examined at a single time point (e.g., delivery/postnatal) [2, 8, 9], thus, do not highlight the true effects of HIV and ART exposure on fetal growth. More accurate measures of fetal growth parameters, such as the velocity and the size of abdominal circumference, biparietal diameter, and head circumference, which are often assessed during routine antenatal clinic visits, have the potential to detect abnormalities *in utero*. In turn, these assessments are more likely to provide rapid and effective intervention strategies, thus preventing adverse birth outcomes and long-term health complications. There is evidence to suggest that HIV and ART treatment [10] and other maternal exposures such as gestational hypertension, gestational weight gain (GWG) and gestational diabetes mellitus (GDM) are associated with poor fetal growth outcomes, particularly the size and/or growth velocity of the head circumference, abdominal circumference and biparietal diameter in a Black South African cohort [11, 12]. Sex differences have been reported, with male fetuses found to be more vulnerable to the adverse effects of gestational hypertension and GDM, whereas female fetuses were more vulnerable to the negative effects of GWG [11, 12].

While the detrimental effects of maternal exposures on fetal development are known, studies accounting for the mediating role of the placenta in these associations are lacking. The placenta plays an essential role as the materno-fetal interface, by acting as a barrier to protect the fetus from maternal toxins and infections [13–16]; and as a transporter of nutrients and essential compounds, which are critical for fetal development [13–16]. To regulate nutrient transfer capacity, or to alleviate distress in a disease state, the placenta adapts morphologically and/or functionally by altering its size, shape, or efficiency in order to maintain appropriate fetal growth [17]. Similarly, umbilical cord length and diameter are important indicators of fetal growth and health. Umbilical cords connect the developing fetus to the placenta, enabling efficient delivery of nutrients, waste elimination and gas exchange from the mother to the fetus [18–20]. Abnormal umbilical cord and length are linked to adverse outcomes such as intrauterine growth restriction, central nervous system defects, and placental pathologies [18–20]. Placentas that deviate from the typical round/oval shape have been associated with adverse outcomes such as SGA [21]. Placental efficiency, defined as the ratio of infant birth weight to placental weight, serves as a measure of placental development and function and plays an important role in maintaining fetal nutritional demands [22]. Both low and high extremes levels of placental efficiency have been linked with disease risk [22]. Thus, the assessment of placental morphology and/or function serves as an important indicator of the changes that occurred *in utero* [14, 15, 23]. Several emerging studies from high-income countries (e.g., Canada) and low- and middle-income countries (e.g., SA, Uganda, Kenya, and Brazil) have provided evidence linking HIV infection and different types of ART regimens (e.g., Protease inhibitors, non-nucleoside reverse transcriptase inhibitors, and integrase inhibitors) to abnormalities in placental morphology and function [15, 16, 24, 25]. Accordingly, HIV and ART exposure was associated with low placental weight and area, and irregular shape [15, 23]. Furthermore, HIV and ART exposure has also been linked to maternal vascular malperfusion [24], an antiangiogenic state [13], and alterations to placental transporters [16], some of which were associated with placental growth disorders [13–16].

To our knowledge, no study has examined the mediating effects of placental morphology on the association between HIV and ART exposure, and longitudinal fetal growth parameters. Hence, this study used structural equation models (SEMs) to examine the impact of HIV and ART exposure on fetal growth outcomes and whether these associations are mediated by placental morphology in urban-dwelling Black South African women. With emerging studies reporting sex differences in response to maternal exposures [11, 12, 26], we also investigated the effects of HIV and ART exposure on fetal growth and placental morphology according to fetal sex.

## Methods

### Study setting and participants

This prospective cohort longitudinal pregnancy study included 372 pregnant women who were the part of the Soweto First 1000 Days (S1000) cohort. Briefly, between 2013 and 2016 pregnant women from the Antenatal Clinic and Fetal Medicine Unit at Chris Hani Baragwanath Academic Hospital (CHBAH) in Soweto, Johannesburg, were invited to the South African Medical Research Council (SA MRC)/Wits Developmental Pathways for Health Research Unit (DPHRU) to participate in the S1000 study. The aim of the S1000 study was to elucidate the complex maternal biological and psycho-social factors that influence fetal and infant growth within the first 1000 days, from conception to two years of age, in an urban African population [27]. The inclusion criteria were: (i) Black (self-reported ethnicity) SA who resided in Soweto; (ii) 18 years of age or older; (iii) preferably < 14 weeks, but no more than 20 weeks, pregnant with a singleton, and naturally conceived pregnancy; (iv) no known diagnosis of epilepsy or diabetes at the time of recruitment; (vi) at least one fetal growth measure of abdominal circumference, biparietal diameter, head circumference, and femur length. Data were collected at six-time points during pregnancy (< 14 weeks; 14–18 weeks; 19–23 weeks; 24–28 weeks; 29–33 weeks and 34–38 weeks), as well as at delivery. Of those invited, 1017 women were recruited into the S1000 cohort. Since placental morphology was not a primary outcome of the S1000 study, only a subset of women (n = 372) had complete data to address our current research question (i.e., maternal HIV and ART exposure, fetal growth measures, and placental morphology variables).

This study was approved by the University of the Witwatersrand’s Human Research Ethics Committee (M120524 and M130309). Participants provided written informed consent before their inclusion in the study.

### Fetal ultrasonography and longitudinal modelling of fetal growth size and velocity using SITAR analytics: outcome variables

All participants had a pregnancy dating scan at the first visit using a Philips HD-9 ultrasound machine (Bothell, Washington, USA). This involved the measurement of the fetal crown–rump length (cm) at < 14 weeks, or the biparietal diameter (diameter across the fetuses head, from one parietal bone to the other; cm), head circumference (cm), and femur length (cm) in more advanced pregnancies (> 14 but < 20 weeks). Participants were invited for follow-up scans at the following visits: 14–18, 19–23, 24–28, 29–33, and 34–38 weeks gestation. Gestational age at each visit, and delivery, was calculated based on the initial dating scan [28]. Abdominal circumference, biparietal diameter, head circumference, and femur length were recorded at each follow-up scan. This methodology was performed as per the INTERGROWTH-21st study international standards for measuring fetal growth [29]. All scans underwent external inter-rater reliability quality assessment at Oxford University as per the INTERGROWTH-21st study standards [30].

Longitudinal fetal growth was modelled using Super Imposition by Translation and Rotation (SITAR). All five serial measurements of each fetal growth parameter (head circumference abdominal circumference, biparietal diameter and femur length) from the first to the third trimester of pregnancy were included in the analyses. A detailed description of the motivation behind the use of SITAR, as well as the modelling methods and results for the study population are presented elsewhere [10]. Briefly, SITAR is a shape-invariant model with a single fitted curve that summarises individual growth patterns according to three parameters [31]– the size, tempo, and velocity of growth [31]. We examined the model fit thoroughly, and for the current analyses, only the size and velocity parameters were obtained. The size parameter represents individual variation along the y-axis, providing an absolute deviation of each individual from the sample mean in the units of the measurement. The velocity parameter represents the contraction or expansion of the individual growth curve relative to the mean curve, providing an indication of the rate of change per unit of time. Males and females were modelled together, and sex differences were assessed by including the sex variable in the model. Data were modelled using the SITAR version 1.0.10 in R version 3.4.2.

### Maternal health characteristics: exposure variables

Per SA’s national PMTCT guidelines, routine HIV counselling and testing are required at the first antenatal visit and at every visit during pregnancy, at delivery, and during breastfeeding [32, 33]. HIV testing is performed using serology testing and viral load assessment [32, 33]. Pregnant women who test positive for HIV are also screened for tuberculosis, sexually transmitted infections, and liver function (alanine aminotransferase) before initiating ART for PMTCT [32, 33]. This information is further recorded on the pregnant woman’s antennal clinic card. All women living with HIV (WLWH) were receiving an ART regimen consisting of efavirenz plus tenofovir plus emtricitabine provided as a fixed dose combination unless contraindicated according to PMTCT guidelines [32, 33]. Therefore, all WLWH were receiving ART during the study and were stratified according to whether they had been initiated on ART prior to pregnancy (pre-pregnancy ART) or during the pregnancy (antenatal ART).

### Maternal anthropometry, demographic and pregnancy-related factors: confounder variables

Maternal anthropometry was collected at the first pregnancy visit. Maternal height (at recruitment) and weight (both at recruitment and each subsequent pregnancy visit) were measured using a stadiometer and digital scale. Weight at recruitment (< 14 weeks) was used as a proxy for pre-pregnancy weight, and together with height, was used to calculate body mass index (BMI = weight [kg]/height [m^2^]). We calculated GWG (kg/week) as [(weight at final pregnancy visit − weight at recruitment)/weeks of follow-up]. Questionnaires, administered via personal interview, were used to collect maternal demographic and household socio-economic status (SES), pregnancy-related and lifestyle factors at recruitment. Parity was defined as the number of previous births (0, 1 or ≥ 2 previous births), at a gestational age of 24 weeks or more – regardless of whether the infant was born alive or was stillborn. Household SES was assessed using an extensively utilised asset index which scored participants according to their possession of 11 possible assets (e.g., car, television, car) [34, 35]. Education was categorized by grades passed. At baseline, smoking status and alcohol use were assessed as current use of cigarettes and/or tobacco and current alcohol use, respectively.

### Birth outcomes: confounder variables

Birth weight and length were measured by trained research nurses within 24 h of delivery. At delivery, the neonate’s sex was recorded, as was the mode of delivery (e.g., vaginal or caesarean). The International Newborn Size at Birth Standards Application tool was used to calculate birth weight centiles, as well as weight-to-length z-scores, according to newborn sex and gestational age at delivery (total days) [36]. The following criteria were used to classify newborns according to their birth weight: small-for-gestational age, < 10th centile; appropriate-for- gestational age, 10th to 90th centile; large-for-gestational age, > 90th centile. Apgar score was taken from the physician delivery report (graded according to neonate skin colour, heart rate, reflexes, muscle tone, and breathing), and scored as above or below 8/10.

### Placenta morphology data: mediator variables

Placentas were collected within 1 h of delivery by research nurses and transferred to the laboratory for processing and storage by laboratory technicians. Excess blood was removed, and the umbilical cord, including separated sections of the cord were laid flat on a board in the field of view of the camera. Metric rulers were placed at right angles to the placenta. Photographs were taken of both the maternal and fetal surfaces. The 2-dimensional size (width and length) of the placenta, umbilical cord length and diameter were later measured digitally using the images and the rulers. The placenta was trimmed of membrane and umbilical cord and weighed using a metric scale. Placental weight to birth weight ratio was calculated to represent placental efficiency. Placental roundness was calculated by subtracting the width from the length of the placenta.

## Statistical analysis

**Fig. 1:**
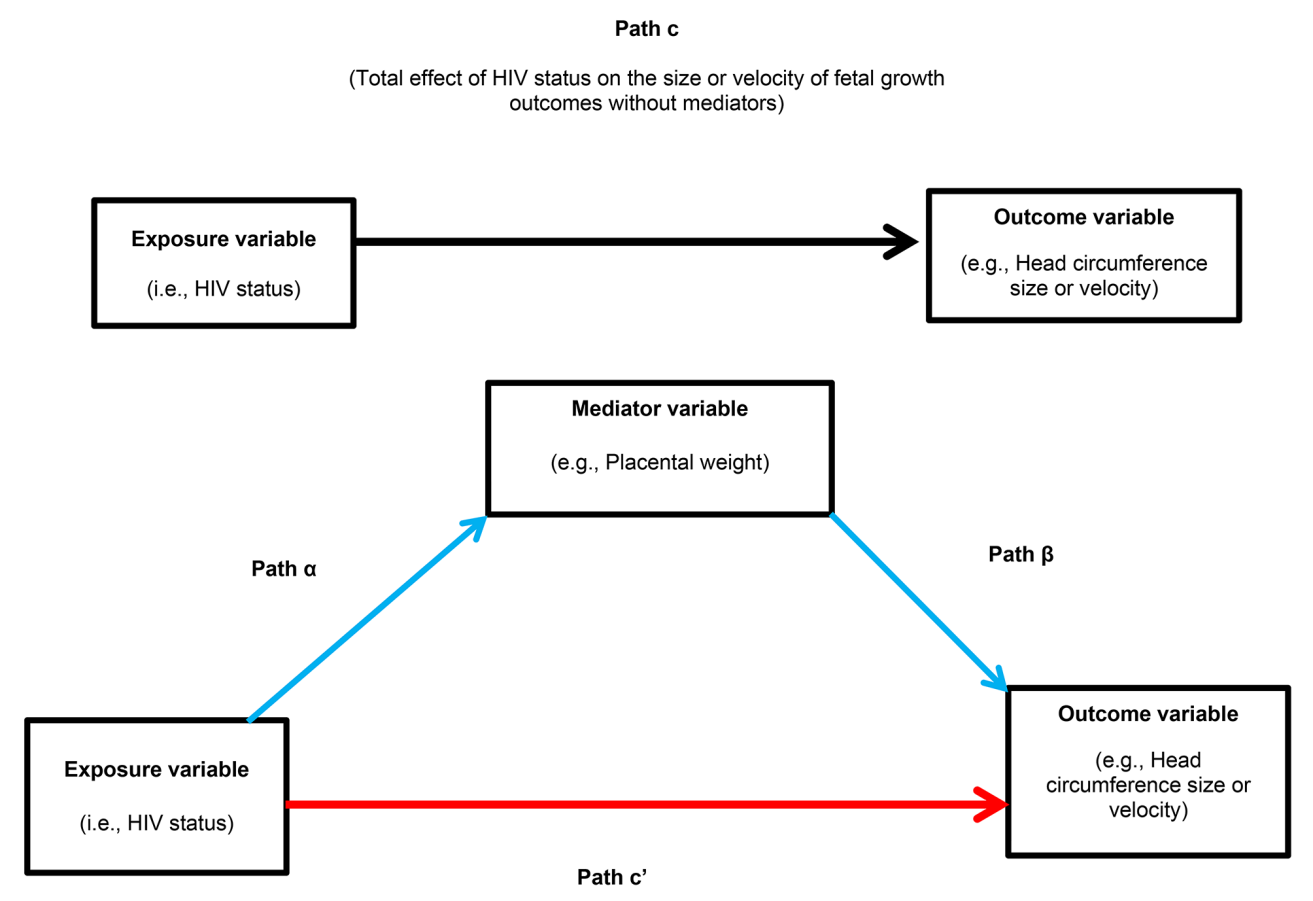
Represents a single SEM mediation analysis model used to examine the effect of HIV status on the size and velocity of fetal growth parameters with placental morphology variables as mediators

The Shapiro-Wilk test was used to assess the distribution of continuous variables. Continuous data are presented as mean (standard deviation, SD) or medians (interquartile range, IQR), and categorical data are presented as frequency (%). A student’s t test or the Wilcoxon rank-sum (Mann-Whitney U test), and the Chi square test was used to examine differences in maternal characteristics, fetal growth parameters, placental morphology outcomes, including birth characteristics between the two groups (WNLWH and WLWH). The variance ratio test was performed to test for equal variance between the groups. A structural equation modelling (SEM) was used to determine whether maternal HIV and ART status (exposure variable) was associated with the size and velocity of the fetal growth parameters (i.e., head and abdominal circumference, biparietal diameter, and femur length) (outcome variables), and whether these associations were mediated by placental morphology variables (i.e., placental weight, efficiency and roundness) (mediator variables) (Fig. 1).

In each mediation model, exposure, outcome and mediator variables were included in order to calculate the unstandardized regression coefficients and generate three outputs: total (i.e., path c), direct (i.e., path c’) and indirect effects (product of paths α and β, αβ) (Fig. 1). Briefly, path c represents the total effect of HIV and ART exposure on the size or velocity of fetal growth parameters without adjusting for mediators. Path α represents the regression coefficient between the exposure and the mediator variables. The regression coefficient of path β represents the effect of the mediator on the size or velocity of the fetal growth outcome. The product of regression coefficients α and β (αβ) represents the mediated (indirect) effect of HIV exposure on the size or velocity of the fetal growth outcome through the mediator variable (Fig. 1). Path c’ represents the direct effect of HIV and ART exposure on the size or velocity of the fetal growth outcome after controlling for the effect of the mediator. Single mediation analyses (one mediator variable per model) were completed to test the effects of HIV and ART exposure on the size or velocity of the fetal growth parameter outcome. Furthermore, two results were generated for each mediation analysis in which the results were stratified by sex. The second part of the results was performed by analysing male and female fetuses together, “combined models”. Models were adjusted for the potential effects of confounders, namely, gestational age at delivery, fetus sex (i.e., in the analyses where male and female fetuses were combined), maternal age, GWG, parity and education status. These confounders were chosen based on previous studies in this setting [8–11]. All data were analysed using Stata® (Version 17.0, StataCorp, College Station, TX, USA). A test was considered statistically significant if *P* ≤ 0.05.

## Results

### Maternal characteristics

The rate of missingness in descriptive study characteristics is presented in Additional Table S1. Rate of missingness in key outcome, exposure, and mediator variables ranged from 0.3 to 2.2% (Additional Table S1). The characteristics of WNLWH (n = 250) and WLWH (n = 122) are presented in Table 1. The WLWH were significantly older (33 (28–37) vs. 28 (25–32) years) and had a lower GWG (0.3 (0.2–0.4) vs. 0.4 (0.3–0.5) kg/week) than WNLWH (*P* < 0.001). Parity and smoking prevalence were higher, while the level of tertiary education was lower in WLWH than WNLWH. There were no significant differences in health characteristics between the two groups. Most WLWH (78%) initiated ART during pregnancy as opposed to pre-pregnancy (Table 1). Moreover, WLWH delivered earlier and had lower placental weights than WNLWH, but these were not statistically different. In contrast, WLWH had a shorter umbilical cord length than WNLWH counterparts (Table 1).

**Table 1:** Maternal characteristics according to HIV status

	N	Total (n = 372, 100%)	WNLWH (n = 250, 67%)	WLWH(n = 122, 33%)	*P*-value
Maternal age, years	372	29 (25–35)	28 (25–32)	33 (28–37)	< 0.001
**Time of enrolment, n(%)**	372				
2013		12 (3.2)	8 (3.2)	4 (3.3)	
2014		151 (40.6))	102 (40.8)	49 (40.2)	0.993
2015		209 (56.2)	140 (56.0)	69 (56.6)	
2016		0	0	0	
**Anthropometry**					
Weight (kg)	372	68.0 (60.0-78.4)	67.6 (59.6–78.2)	69.4 (60.2–79.3)	0.306
Height (cm)	372	159 (155–163)	159 (154–163)	158 (156–162)	0.955
BMI at recruitment (kg/m^2^) (< 14 weeks)	372	27.0 (23.7–30.7)	26.8 (23.5–30.5)	27.2 (24.0-31.2)	0.327
GWG (kg/week)	371	0.4 (0.2–0.5)	0.4 (0.3–0.5)	0.3 (0.2–0.4)	< 0.001
**Demographic and socioeconomic characteristics**					
Parity	372				
Para 0		85 (22.8)	68 (27.2)	17 (13.9)	0.013
Para 1		256 (68.8)	164 (65.6)	92 (75.4)	
Para ≥ 2		31 (9.3)	18 (7.2)	13 (10.7)	
SES asset index score (score/11)	372	5 (5–6)	6 (5–6)	5 (5–6)	0.123
Maternal education, highest level achieved	372				
Primary		12 (3.2)	8 (3.2)	4 (3.2)	0.025
Secondary		270 (72.6)	171 (68.4)	99 (81.2)	
Tertiary		90 (24.2)	71 (28.4)	19 (15.6)	
**Lifestyle characteristics**					
Alcohol use during pregnancy (yes)	317	31 (9.8)	18 (8.3)	13 (12.9)	0.205
Smoking use during pregnancy	255	27 (7.3)	13 (5.2)	14 (11.5)	0.029
**Maternal health characteristics**					
HIV treatment	122				
Pre-pregnancy ART initiation				27 (22.1)	
Antenatal ART initiation				95 (77.9)	
HIV treatment (< 2 or 2 years or more)	122				
ART < 2 years				111 (91.0)	
ART ≥ 2 years or more				11 (9.0)	
GDM (yes)	337	43 (12.8)	25 (10.9)	18 (16.8)	0.127
Anaemia (yes)	372	134 (35.6)	90 (39.0)	44 (36.1)	0.990
Hypertension (yes)	372	31 (8.2)	17 (6.8)	10 (8.2)	0.626
**Birth outcomes**					
**Neonate sex**	372				
Male		197 (53.0)	138 (55.2)	59 (48.4)	0.215
Female		175 (47.0)	112 (44.8)	63 (51.6)	
Gestational age at time of enrolment (days)	372	88 (79–95)	89 (79–94)	88 (78–96)	0.930
Gestational age at delivery (weeks)	372	38 (37–39)	39 (37–40)	38 (37–39)	0.050
Mode of delivery	372				
Vaginal		160 (43.0)	111 (44.4)	49 (40.2)	0.438
Caesarean section		212 (57.0)	139 (55.6)	73 (59.8)	
**Anthropometry**					
Birth weight (kg)	369	3.0 (2.6–3.2)	3.0 (2.7–3.2)	2.9 (2.4–3.2)	0.091
Birth weight category^a^	369				
Small-for-gestational age		67 (18.1)	39 (15.8)	28 (23.0)	0.244
Appropriate-for-gestational age		280 (75.9)	193 (78.1)	87 (71.3)	
Large-for-gestational age		22 (6.0)	15 (6.1)	7 (5.7)	
Head circumference (cm)	369	34 (33–35)	34 (33–35)	34 (33–35)	0.135
Birth length (cm)	369	48 (46–49)	48 (47–50)	48 (45–49)	0.058
Apgar Score (> 8); n(%)	369	351 (95.1)	232 (93.9)	119 (97.5)	0.129
**Placental parameters**					
Placental weight (g)	367	451.3 (386.2-520.2)	462.5 (394.7-530.7)	436.7 (377.8-496.6)	0.061
Placental efficiency	364	6.4 (5.7–7.3)	6.4 (5.7–7.3)	6.5 (5.7–7.4)	0.616
Placental length (cm)	371	20.0 (18.6–21.4)	20.0 (18.7–21.3)	20.0 (18.5–21.5)	0.840
Placental width (cm)	370	18.2 (16.8–19.5)	18.2 (17.0-19.5)	18.2 (16.5–19.6)	0.979
Placental roundness (cm)	368	1.4 (0.7–2.5)	1.4 (0.7–2.5)	1.5 (0.8–2.6)	0.461
Umbilical cord length (cm)	366	30.0 (24.0-36.5)	31.3 (25.0-37.8)	27.3 (21.7–33.3)	0.002
Umbilical cord diameter (cm)	364	1.1 (1.0-1.3)	1.1 (1.0-1.3)	1.2 (1.0-1.4)	0.383

Continuous data are presented as median (IQR) and categorical data presented as n (%). ART, Antiretroviral therapy; BMI, Body mass index; GDM, Gestational diabetes mellitus; GWG, Gestational weight gain; HIV, Human immunodeficiency virus; SES, Socioeconomic status. WLWH, Women living with HIV; WNLWH, Women not living with HIV. ^a^Calculated using the International Newborn Size at Birth Standards Application tool [36]

Birth, anthropometric and placental morphology outcomes of fetuses exposed and unexposed to HIV and ART and were sex-stratified and compared in Table 2. Only umbilical cord length was significantly shorter in male fetuses of WLWH than in male fetuses of WNLWH (27.3 (21.6–32.8) cm vs. 31.4 (25.0–37.0) cm, *P* = 0.015). Similarly, female fetuses born to WLWH had shorter umbilical cord lengths than female fetuses born to WNLWH (28.6 (22.2–34.1) vs. 31.0 (25.0-37.8) cm, *P* = 0.051). Furthermore, gestational age at delivery, birth weight, head circumference, birth length and placental weight were significantly lower in female fetuses of WLWH than their counterparts (Table 2).

**Table 2:** Characteristics of male and female fetuses according to maternal HIV status

	Male (n = 197, 53%)			Female (n = 175, 47%)		
	Unexposed to HIV (n = 138, 70%)	Exposed to HIV (n = 59, 30%)	P-value	Unexposed to HIV (n = 112, 64%)	Exposed to HIV (n = 63, 36%)	*P*-value
**Birth outcomes**						
Gestational age at delivery (weeks)	38 (37–39)	38 (37–39)	0.385	39 (38–40)	38 (37–39)	0.042
Mode of delivery						
Vaginal	59 (42.8)	20 (33.9)	0.245	52 (46.4)	29 (46.0)	0.960
Caesarean section	79 (57.2)	39 (66.1))		60 (53.6)	34 (54.0)	
**Anthropometry**						
Birth weight (kg)	3.0 (2.7–3.3)	3.0 (2.6–3.3)	0.945	3.0 (2.7–3.2)	2.9 (2.3–3.1)	0.019
Birth weight category^a^						
Small-for-gestational age	23 (16.9)	12 (20.3)	0.839	16 (14.1)	16 (25.4)	0.207
Appropriate-for-gestational age	106 (77.9)	44 (74.6)		87 (78.4)	43 (68.3)	
Large-for-gestational age	7 (5.2)	3 (5.1)		8 (7.2)	4 (6.3)	
Head circumference (cm)	34 (33–35)	34 (33–35)	0.576	34 (33–35)	33 (32–34)	0.005
Birth length (cm)	48 (46–50)	48 (46–50)	0.641	48 (47–49)	47 (45–49)	0.022
Apgar Score (> 8); n(%)	125 (92.6)	57 (96.6)	0.285	107 (95.5)	62 (98.4)	0.315
**Placental parameters**						
Placental weight (g)	454.4 (373.8-532.4)	439.0 (357.1-493.2)	0.823	466.8 (415.8-525.7)	435.2 (384.9–500.0)	0.022
Placental efficiency	6.4 (5.8–7.4)	6.5 (5.8–7.8)	0.544	6.3 (5.6–7.1)	6.5 (5.6–7.1)	0.867
Placental length (cm)	19.9 (18.5–21.3)	19.8 (18.4–22.0)	0.768	20.0 (18.8–21.4)	20.0 (18.7–21.4)	0.975
Placental width (cm)	18.2 (17.1–19.5)	18.1 (16.5–19.6)	0.795	18.1 (16.7–19.5)	18.5 (16.5–19.9)	0.794
Placental roundness (cm)	1.3 (0.7–2.5)	1.5 (0.7–2.6)	0.474	1.4 (0.6–2.9)	1.4 (0.9-3.0)	0.783
Umbilical cord length (cm)	31.4 (25.0–37.0)	27.3 (21.6–32.8)	0.015	31.0 (25.0-37.8)	28.6 (22.2–34.1)	0.051
Umbilical cord diameter (cm)	1.2 (1.0-1.4)	1.3 (1.0-1.4)	0.397	1.1 (1.0-1.3)	1.1 (1.1–1.3)	0.512

Continuous data are presented as median (IQR) and categorical data presented as %. ART, Antiretroviral therapy; HIV, Human immunodeficiency virus. ^a^Calculated using the International Newborn Size at Birth Standards Application tool [36]

### Fetal growth outcomes

**Fig. 2:**
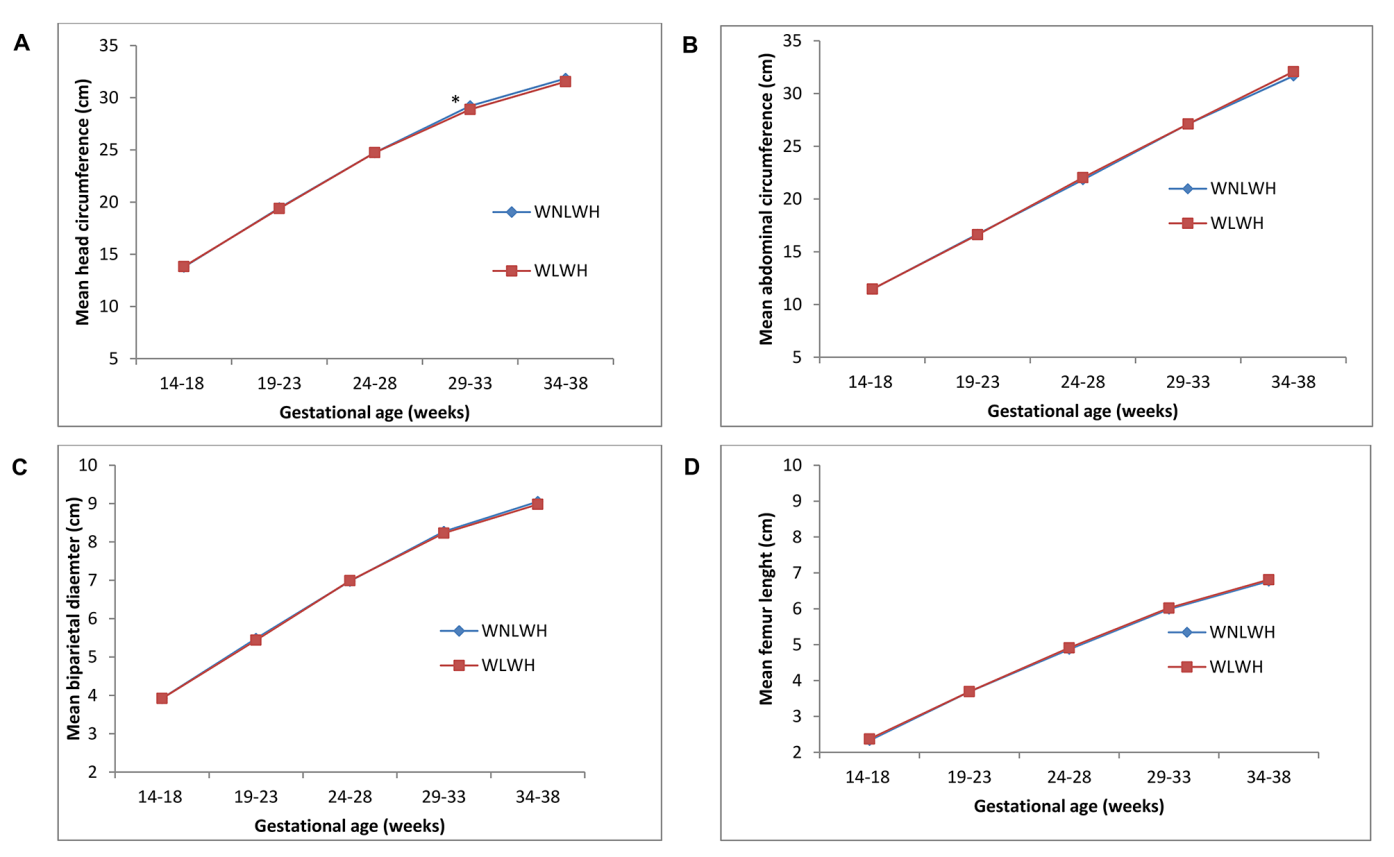
Head circumference (A), abdominal circumference (B), biparietal diameter (C) and femur length (D) of fetuses born to WLWH (n = 122; red line) and WNLWH (n = 250; blue) against gestational age in weeks

Overall, fetal growth parameters were similar between the two groups throughout pregnancy (Fig. 2 and Table S2). Only head circumference was significantly lower in fetuses of WLWH than fetuses of WNLWH, particularly at visit 5 (Fig. 2). Even after stratification by sex, head circumferences at visits 5 and 6, including biparietal diameter at visit 6 were significantly lower in female fetuses of WLWH than their counterparts (Table S3). In contrast, no differences between male fetuses were present, and no other fetal growth measures were different when stratified by sex.

### SEMs for the effects of HIV exposure on the size and velocity of fetal growth outcomes through placental morphology mediators

The total, direct, and indirect effects of HIV and ART exposure on fetal growth outcomes are presented in Table 3 (size of fetal growth outcomes) and Table 4 (velocity of fetal growth outcomes). When stratified by sex, HIV and ART exposure was inversely associated with the size and growth velocity of head circumference in females only (Tables 3 and 4). This association remained significant even after controlling for the potential mediating effects of the placenta (Tables 3 and 4). In male fetuses, HIV and ART exposure was positively associated with femur length size and velocity before and after adjusting for placental weight and roundness (Tables 3 and 4). Moreover, a positive association was found between HIV and ART exposure and abdominal circumference velocity in male fetuses (Table 4). There were no significant associations between HIV and ART exposure and other measures of fetal growth when stratified by sex. Notably, HIV and ART exposure had no significant total, direct, or indirect effects on the size and velocity of fetal growth outcomes when male and female fetuses were analysed together (Tables S4 and S5).

**Table 3:** Effect of HIV exposure on the size of fetal growth outcomes, with placental morphology variables as mediators

	Effect of HIV exposure on:	Total effects (Path c)		Direct effects (Path c’)		Indirect effects (Product of paths α and β, αβ)	
		Estimate (95% CI)	*P*-value	Estimate (95% CI)	*P*-value	Estimate (95% CI)	*P*-value
	**Head circumference (size)**						
Male	Without placental weight	**0.156 (-0.075-0.387)**	**0.185**				
	Via placental weight			0.156 (-0.068-0.379)	0.172	0.000 (-0.058-0.059)	0.988
	Without placental efficiency	**0.144 (-0.088-0.375)**	**0.244**				
	Via placental efficiency			0.142 (-0.088-0.373)	0.226	0.002 (-0.024-0.028)	0.902
	Without placental roundness	**0.157 (-0.078-0.392)**	**0.191**				
	Via placental roundness			0.150 (-0.084-0.385)	0.210	0.007 (-0.015-0.029)	0.544
Female	Without placental weight	**-0.317 (-0.527- -0.108)**	**0.003**				
	Via placental weight			-0.299 (-0.507- -0.091)	0.005	-0.019 (-0.054-0.0169)	0.302
	Without placental efficiency	**-0.334 (-0.539- -0.129)**	**0.001**				
	Via placental efficiency			-0.341 (-0.545- -0.137)	0.001	0.007 (-0.016-0.031)	0.552
	Without placental roundness	**-0.319 (-0.525- -0.113)**	**0.002**				
	Via placental roundness			-0.322 (-0.527- -0.115)	0.002	0.003 (-0.010-0.015)	0.600
	**Abdominal circumference (size)**						
Male	Without placental weight	**0.199 (-0.069-0.466)**	**0.145**				
	Via placental weight			0.198 (-0.046-0.442)	0.111	0.001 (-0.110-0.111)	0.988
	Without placental efficiency	**0.163 (-0.097-0.423)**	**0.220**				
	Via placental efficiency			0.163 (-0.097-0.423)	0.220	-0.000 (-0.002-0.002)	0.985
	Without placental roundness	**0.233 (-0.034-0.500)**	**0.087**				
	Via placental roundness			0.234 (-0.032-0.502)	0.086	-0.001 (-0.016-0.013)	0.841
Female	Without placental weight	**-0.054 (-0.343-0.234)**	**0.711**				
	Via placental weight			-0.001 (-0.278-0.276)	0.994	-0.053 (-0.142-0.035)	0.238
	Without placental efficiency	**-0.067 (-0.354-0.221)**	**0.648**				
	Via placental efficiency			-0.071 (-0.360-0.217)	0.628	0.004 (-0.015-0.0.024)	0.663
	Without placental roundness	**-0.035 (-0.328-0.257)**	**0.813**				
	Via placental roundness			-0.031 (-0.323-0.261)	0.836	-0.004 (-0.024-0.015)	0.663
	**Biparietal diameter (size)**						
Male	Without placental weight	**0.052 (-0.025-0.130)**	**0.186**				
	Via placental weight			0.052 (-0.023-0.128)	0.175	0.000 (-0.018-0.018)	0.988
	Without placental efficiency	**0.048 (-0.030-0.126)**	**0.223**				
	Via placental efficiency			0.048 (-0.030-0.125)	0.226	0.000 (-0.009-0.010)	0.902
	Without placental roundness	**0.052 (-0.026-0.131)**	**0.191**				
	Via placental roundness			0.050 (-0.028-0.128)	0.211	0.002 (-0.005-0.010)	0.536
Female	Without placental weight	**-0.042 (-0.113-0.029)**	**0.245**				
	Via placental weight			-0.036 (-0.106-0.035)	0.323	-0.006 (-0.019-0.006)	0.297
	Without placental efficiency	**-0.046 (-0.117-0.024)**	**0.199**				
	Via placental efficiency			-0.049 (-0.119-0.021)	0.169	0.003 (-0.006-0.012)	0.541
	Without placental roundness	**-0.049 (-0.120-0.021)**	**0.173**				
	Via placental roundness			-0.049 (-0.120-0.021)	0.176	-0.000 (-0.003-0.003)	0.831
	**Femur length (size)**						
Male	Without placental weight	**0.064 (0.003–0.126)**	**0.041**				
	Via placental weight			0.064 (0.004–0.124)	0.037	0.000 (-0.012-0.012)	0.988
	Without placental efficiency	**0.055 (-0.004-0.115)**	**0.067**				
	Via placental efficiency			0.055 (-0.004-0.115)	0.068	0.000 (-0.005-0.006)	0.902
	Without placental roundness	**0.064 (0.001–0.126)**	**0.045**				
	Via placental roundness			0.063 (0.000-0.126)	0.047	0.001 (-0.003-0.004)	0.737
Female	Without placental weight	**-0.010 (-0.072-0.052)**	**0.741**				
	Via placental weight			-0.005 (-0.067-0.057)	0.871	-0.005 (-0.0156-0.005)	0.309
	Without placental efficiency	**-0.014 (-0.075-0.047)**	**0.650**				
	Via placental efficiency			-0.016 (-0.078-0.045)	0.596	0.002 (-0.005-0.010)	0.547
	Without placental roundness	**-0.014 (-0.075-0.047)**	**0.651**				
	Via placental roundness			-0.015 (-0.076-0.045)	0.613	0.001 (-0.004-0.007)	0.608

Adjusted for age for the potential effects of fetal sex (only in the combined models), gestational age at delivery, maternal age, GWG, parity and education. GWG, Gestational weight gain; HIV, Human immunodeficiency virus

**Table 4:** The effects of HIV exposure on the velocity of fetal growth outcomes, with placental morphology measures as mediators

	Effect of HIV exposure on:	Total effects (Path c)		Direct effects (Path c’)		Indirect effects (Product of paths α and β, αβ)	
		Estimate (95% CI)	*P*-value	Estimate (95% CI)	*P*-value	Estimate (95% CI)	*P*-value
	**Head circumference (velocity)**						
Male	Without placental weight	**0.006 (-0.008-0.020)**	**0.382**				
	Via placental weight			0.006 (-0.007-0.020)	0.366	0.000 (-0.003-0.004)	0.988
	Without placental efficiency	**0.005 (-0.009-0.020)**	**0.467**				
	Via placental efficiency			0.005 (-0.009-0.019)	0.473	0.000 (-0.001-0.001)	0.902
	Without placental roundness	**0.006 (-0.008-0.020)**	**0.412**				
	Via placental roundness			0.006 (-0.008-0.020)	0.428	0.000 (-0.000-0.001)	0.670
Female							
	Without placental weight	**-0.022 (-0.035- -0.008)**	**0.001**				
	Via placental weight			-0.021 (-0.034- -0.007)	0.002	-0.009 (-0.003-0.001)	0.340
	Without placental efficiency	**-0.023 (-0.036- -0.009)**	**0.001**				
	Via placental efficiency			-0.023 (-0.036- -0.010)	0.000	0.000 (-0.001-0.002)	0.545
	Without placental roundness	**-0.022 (-0.035- -0.008)**	**0.001**				
	Via placental roundness			-0.022 (-0.035- -0.008)	0.001	0.000 (-0.001-0.001)	0.665
	**Abdominal circumference (velocity)**						
Male	Without placental weight	**0.018 (-0.003-0.039)**	**0.092**				
	Via placental weight			0.018 (-0.001-0.037)	0.064	0.000 (-0.008-0.009)	0.988
	Without placental efficiency	**0.015 (-0.005-0.036)**	**0.143**				
	Via placental efficiency			0.015 (-0.005-0.036)	0.142	-0.000 (-0.000-0.000)	0.907
	Without placental roundness	**0.021 (0.000-0.042)**	**0.050**				
	Via placental roundness			0.021 (0.000-0.042)	0.047	-0.000 (-0.006-0.001)	0.675
Female	Without placental weight	**-0.004 (-0.026-0.019)**	**0.756**				
	Via placental weight			0.000 (-0.021-0.022)	0.946	-0.004 (-0.011-0.003)	0.236
	Without placental efficiency	**-0.004 (-0.027-0.018)**	**0.697**				
	Via placental efficiency			-0.004 (-0.027-0.018)	0.681	0.000 (-0.001-0.001)	0.719
	Without placental roundness	**-0.001 (-0.024-0.022)**	**0.929**				
	Via placental roundness			-0.001 (-0.024-0.022)	0.945	-0.000 (-0.001-0.001)	0.715
	**Biparietal diameter (velocity)**						
Male	Without placental weight	**0.010 (-0.006-0.027)**	**0.238**				
	Via placental weight			0.010 (-0.006-0.027)	0.220	0.000 (-0.004-0.004)	0.988
	Without placental efficiency	**0.009 (-0.008-0.026)**	**0.300**				
	Via placental efficiency			0.009 (-0.008-0.026)	0.304	0.000 (-0.001-0.002)	0.902
	Without placental roundness	**0.009 (-0.007-0.027)**	**0.276**				
	Via placental roundness			0.009 (-0.008—0.027)	0.287	0.000 (-0.001-0.001)	0.707
Female	Without placental weight	**-0.010 (-0.028-0.006)**	**0.237**				
	Via placental weight			-0.009 (-0.026-0.008)	0.293	-0.001 (-0.003-0.001)	0.350
	Without placental efficiency	**-0.011 (-0.028-0.005)**	**0.197**				
	Via placental efficiency			-0.012 (-0.029-0.004)	0.157	0.001 (-0.002-0.003)	0.527
	Without placental roundness	**-0.012 (-0.030-0.005)**	**0.160**				
	Via placental roundness			-0.012 (-0.030-0.005)	0.162	-0.000 (-0.000-0.001)	0.939
	**Femur length (velocity)**						
Male	Without placental weight	**0.011 (0.00-0.022)**	**0.038**				
	Via placental weight			0.011 (0.001–0.022)	0.032	0.000 (-0.002-0.003)	0.988
	Without placental efficiency	**0.010 (-0.006-0.021)**	**0.064**				
	Via placental efficiency			0.010 (-0.006-0.021)	0.064	0.000 (-0.00-0.000)	0.915
	Without placental roundness	**0.011 (0.000-0.022)**	**0.048**				
	Via placental roundness			0.011 (-0.000-0.022)	0.051	0.000 (-0.000-0.001)	0.675
Female	Without placental weight	**-0.004 (-0.017-0.008)**	**0.479**				
	Via placental weight			-0.003 (-0.016-0.009)	0.575	-0.001 (-0.002-0.001)	0.331
	Without placental efficiency	**-0.005 (-0.017-0.007)**	**0.415**				
	Via placental efficiency			-0.005 (-0.017-0.006)	0.385	0.000 (0.000-0.001)	0.577
	Without placental roundness	**-0.005 (-0.017-0.006)**	**0.385**				
	Via placental roundness			-0.006 (-0.018-0.006)	0.333	0.001 (-0.001-0.002)	0.583

Adjusted for age for the potential effects of fetal sex (only in the combined models), gestational age at delivery, maternal age, GWG, parity and education. GWG, Gestational weight gain; HIV, Human immunodeficiency virus

The association between HIV and ART exposure and mediators (Path α) are presented in Table S6, while the associations between mediators and fetal growth outcomes (Path β), are presented in Table S7. There were no significant associations between HIV and ART exposure and placental morphology variables when stratified by sex (Table S6, Path β) or when male and female fetuses were analysed together (Table S6). However, some of the placental morphology variables were significantly associated with the size and velocity of the fetal growth outcomes in the models of male and female fetuses (Path β, Table S7) and in the combined models (Table S7). For instance, placental weight was positively associated with the size and velocity of the fetal growth outcomes in male fetuses. However, neither placental efficiency nor roundness were associated with fetal growth parameters in male fetuses (Table S7). In females, placental weight, efficiency, and roundness were associated with abdominal circumference (both size and velocity), biparietal diameter velocity and femur length velocity, respectively (Table S7).

## Discussion

This study’s main finding was that we did not observe a mediating effect of multiple key placental morphologic features on the relationship of HIV and ART exposure with fetal growth outcomes in urban-dwelling Black South African women. However, we did find that HIV and ART exposure was directly associated with reduced head circumference size and velocity in female fetuses. In male fetuses, HIV and ART exposure was positively associated with femur length growth (both size and velocity) and abdominal circumference velocity. Furthermore, we noted that HIV and ART exposure was not significantly associated with measures of placental morphology such as weight, efficiency, and roundness. Nonetheless, there were some associations between placental morphology and fetal growth outcomes.

Due to its function as a materno-fetal interface, the placenta is believed to be the key mediator in the association between maternal HIV and ART exposure, and adverse health outcomes such as LBW, PTB, SGA [14, 15, 23, 37]. However, none of these studies accounted for the mediating role of the placenta in these associations [14, 15, 23, 37]. Instead, these findings were based on comparisons of placental morphologic features and/or birth outcomes according to HIV and ART status [14, 15, 23, 37] and on the associations of HIV and ART exposure with placental morphology as an outcome variable, or between placental morphology (as an exposure variable) and adverse birth outcomes [14, 15, 23, 37]. Accordingly, HIV and ART exposure was associated with low placental weights, areas and irregular shapes, and some of these placental morphology features were further associated with unfavourable birth outcomes [14, 15, 23, 37]. Similarly, when we compared maternal and fetal characteristics according to HIV and ART status, we showed shorter umbilical cord, lower placental weight, length, birth length, head circumference growth in women-fetus pairs exposed to HIV and ART compared to their counterparts. However, when we included all three variables (i.e., exposure, mediator and outcome variables) in an SEM model and further adjusted for known confounders, we showed that HIV and ART exposure was not significantly associated with placental morphology parameters, and that placental morphology did not mediate the association between HIV and ART exposure and fetal growth. Rather, few direct associations were reported, which included a reduced head circumference growth in females and increased femur length and abdominal growth in male fetuses born to WLWH. The lack of mediation effect by the placental morphology in the association of HIV and ART exposure with fetal growth outcomes, and between the association of HIV and ART exposure and placental morphology is notable and might be attributed to several factors such as the timing of ART initiation, severity and duration of HIV infection and ART exposure, and how well the WLWH were treated. Indeed, pre-conception initiation of ART (vs. ART initiated during pregnancy) and longer duration of HIV exposure (e.g., *in utero* vs. perinatal HIV exposure) have been associated with more adverse developmental and functional outcomes such as reduced head circumference, LWB, PTB, and placental abnormalities [2, 4, 24, 38]. In line with these findings, we showed that the majority of WLWH initiated ART during pregnancy (78.0%), presumably when the diagnosis of HIV was first made. According to previous reports from SA, most women who initiate ART during pregnancy tend to be younger and healthier compared to their counterparts who initiate ART before pregnancy [24, 38]. Our findings could also be contributed to the fact that all women were receiving efavirenz-based regimen, which has fewer adverse effects compared to other regimens (e.g., dolutegravir-based regimen) [39]. Also, due to the fact that WLWH receiving ART had presumably good retention and adherence to ART, and viral suppression. Furthermore, gross placental morphology may not necessarily represent the entire function of the placenta, and its interaction with HIV and ART. We did not examine histological and histopathological correlates of fetal growth measures. It remains possible that microscopic features of tissue morphology and cellular composition could correlate more strongly with, and possibly mediate the association between HIV and ART exposure, and fetal growth measures. Additionally, other biological processes not examined in this study, including drug transporters in the placenta and other fetal tissues such as the liver and blood-brain barriers [16, 40, 41] or the vascular system [13, 14] and metabolic enzymes [41], might have mediated the effects of HIV and ART exposure on fetal growth outcomes. Indeed, multiple drug transporters were altered in the placentas of WLWH [16]. Moreover, changes to drug transporters or metabolic enzymes expressed in the blood-brain barrier may lead to the influx of HIV and ART, thereby targeting the developing brain, which is vulnerable to HIV infection [2, 42, 43]. In rodent models, perinatal exposure to efavirenz was shown to disrupt the cellular composition of the prefrontal cortex and serotoninergic system in male offspring [44, 45]. These alterations were linked to delays in reflex and motor development [44, 45]. In line with these findings, the inverse association between HIV and ART exposure and head circumference in female fetuses may highlight the contribution of these proposed biological mechanisms, in particular the brain systems, as potential alternative mediators in this interaction. Notably, altered head growth in female fetuses may increase the likelihood of neurodevelopmental abnormalities later in life. In line with this notion, several studies have reported lower brain volume, head circumference and scores on neurodevelopment assessments, and increased risk of neurological disorders (i.e., microcephaly) in HIV-positive or HIV-exposed but uninfected children (both males and females) born to mothers who were receiving ART [2, 3, 42, 43], particularly combination regimens of efavirenz [3]. Future studies need to investigate whether the inverse association between HIV and ART exposure and head circumference in female fetuses has any adverse long-term developmental and functional outcomes in a South African cohort.

To our knowledge, this is the first study to report a positive association between HIV and ART exposure and femur length growth. In contrast, Jao, et al. [46] did not find an association between tenofovir exposure and femur and humerus growth in another South African cohort (n = 646) of HIV-exposed but uninfected children. With these contradictory findings, the impact of HIV and ART exposure on bone health remains unclear. Nonetheless, we propose the positive association between HIV and/or ART exposure and femur length in male fetuses might be largely attributed to the protective effects of ART use. In a large cohort of HIV-exposed but uninfected children (n = 3055) who were enrolled in the Surveillance Monitoring for ART Toxicities (SMARTT) study at 22 clinical sites in the USA, including Puerto Rico, it was reported that exposure to darunavir was protective against microcephaly, whereas exposure to combination regimens of efavirenz including tenofovir and emtricitabine was associated with a higher risk of microcephaly [3]. These results suggest that different types of ART regimens might have protective associations with specific growth outcomes in HIV-exposed but uninfected children.

Although male fetuses might be protected against a shorter stature and possibly restricted-head growth, the positive association between HIV and ART exposure and abdominal circumference suggests that this group may develop metabolic disorders later in life. In accordance with earlier reports using the same population, fetal abdominal circumference growth was positively associated with neonatal adiposity and shown to mediate the negative effects of GWG on neonatal adiposity [11]. Moreover, the associations between HIV and ART exposure and fetal growth outcomes are in accordance with previous findings in which other maternal exposures including gestational hypertension, GDM and GWG were associated with fetal growth outcomes in the same sample [10, 12]. As with the current study, sex differences were also reported, where male fetuses were more susceptible to negative growth consequences when mothers had gestational hypertension and/or GDM [10, 12]. However, female fetuses were vulnerable to the negative effects of GWG [10]. Sex differences in response to HIV and ART exposure have also been reported with other adverse health outcomes such as aneamia and cardiovascular defects (e.g., left ventricular structure abnormalities) [47, 48]. Collectively, these findings suggest that males and females are susceptible to different maternal exposures. The exact contributing risk factors to these sex differences are not fully understood but might partly be explained by sex-specific gene expressions, in particular at the sites (i.e., placenta, liver or brain-blood barrier) proposed to mediate the effects of HIV and ART exposure on fetal growth outcomes [41]. The evidence of sex interactions in response to HIV and ART exposure warrants further investigation.

Our study has some limitations in that S1000’s main research question was not specifically related to placental morphology, hence this study was only conducted in a subsample of the S1000 cohort resulting in a limited sample size. Nonetheless, this study allowed us to explore HIV and ART exposure in relation to fetal development and placental morphology. This is a cross-sectional study, thus our results do not imply causality. Future research is needed to explain the effects of HIV and ART exposure on fetal growth outcomes, and whether these associations are associated with long-term developmental and functional abnormalities in a South African population. Furthermore, we were unable to adjust for other potential confounders such as viral load or CD4 count or adherence to ART. Due to the universal guidelines that all pregnant women living with HIV should receive ART, it is difficult to delineate the effects of HIV from those of ART on fetal growth. Therefore, we were unable to conclude whether our findings are due to HIV or treatment, or the combination of both. Given that the sample was recruited between 2013 and 2016 and receiving an efavierenz-based regimen, our findings might not be applicable to other WLWH who are taking different ART regimens (e.g., dolutegravir-based regimens). The assessment of placental morphology was restricted to gross estimates, which may not fully represent placental function. Despite this, our analysis had several advantages. We had HIV and ART-unexposed women and fetuses as comparator groups, which enabled us to explore maternal and fetal characteristics between HIV and ART status; and to test whether the effects of HIV and ART on fetal growth are directly or indirectly mediated by placental morphology. Furthermore, we investigated fetal sex as an independent variable, allowing us to examine sex differences in response to HIV and ART exposure.

## Conclusion

In summary, our findings suggest that HIV and/or ART exposure during pregnancy in urban-dwelling Black South African women may directly impact some growth parameters, and that this interaction might also vary according to fetal sex, yet does not seem to be mediated by placental morphology. Whether these associations are linked to adverse health outcomes across the life course, remains to be fully elucidated. This highlights the complexity of HIV and ART exposure on fetal development. Therefore, it is recommended that exploration of placental drug transporter system and metabolic enzymes in fetus tissues, including sex-specific genes in these sites as potential mediators, might provide better insights into the impact of HIV and ART exposure on fetal growth. More prospective studies are needed to provide better insights on the risks and benefits of different ART regimens (e.g., efavirenz-based vs. dolutegravir regimens), and the timing of ART initiation (pre-conception, early and late pregnancy), particularly in women of reproductive age in high-risk countries such as SA. This knowledge will help clinicians and policymakers in producing safer ART regimes and in formulating new guidelines that also consider fetal sex, types of ART regimens, and timing of ART initiation as important risk factors in determining the health status of the child. This is paramount since the current guidelines are favoring the use of dolutegravir-based regimens as first-line ART due to stronger viral suppression and higher genetic barrier to resistance compared to efavirenz-based regimens [39, 49]. Despite these benefits, little is known about the effects of dolutegravir-based regimens and their associations with placental morphology, fetal growth parameters, and related developmental and functional implication across the life course, especially in a South African population.

## Electronic supplementary material

Below is the link to the electronic supplementary material.

Supplementary Material 1

## Data Availability

The datasets used and/or analysed during the current study are available from the corresponding author on reasonable request.

## Abbreviations

ART: Antiretroviral therapy
BMI: Body mass index
CHBAH: Chris Hani Baragwanath Academic Hospital
GDM: Gestational diabetes mellitus
GWG: Gestational weight gain
HIV: Human immunodeficiency virus
IQR: Interquartile range
LBW: Low birth weight
PMTCT: Prevent mother-to-child transmission
PTB: Preterm birth
SA: South Africa
SA MRC/Wits DPHRU: South African Medica Research Council/Wits Developmental Pathways for Health Research Unit
SD: Standard deviation
SEM: Structural equation model
SES: Socioeconomic status
SGA: Small-for-gestational age
SITAR: Super imposed by translation and rotation
USA: United States of America
WLWH: Women living with HIV
WNLWH: Women not living with HIV
