# Supplementary material for: The impact of HIV and ART exposure during pregnancy on fetal growth: a prospective study in a South African cohort
Source: BMC Pregnancy Childbirth. 2023 Jun 3;23:415. doi: 10.1186/s12884-023-05743-x (PMC10239583; doi:10.1186/s12884-023-05743-x)
Supplement: Supplementary file 1 — Supplementary Material 1 [file 12884_2023_5743_MOESM1_ESM.pdf]

Supplementary Materials

Table S1 Rate of missingness of study characteristics

	**N**	**Rate of missingness (%)**
Maternal age, years	372	0
**Time of enrolment, n(%)**	372	0
2013		
2014		
2015		
2016		
**Anthropometry**		
Weight (kg)	372	0
Height (cm)	372	0
BMI at recruitment (kg/m^2) (<14 weeks)	372	0
GWG (kg/week)	371	0.3
**Demographic and socioeconomic characteristics**		
Parity	372	0
Para 0		
Para 1		
Para ≥ 2		
SES asset index score (score/11)	372	0
Maternal education, highest level achieved	372	0
Primary		
Secondary		
Tertiary		
**Lifestyle characteristics**		
Alcohol use during pregnancy (yes)	317	14.8
Smoking use during pregnancy	255	31.4
**Maternal health characteristics**		
HIV treatment	122	0
Antenatal ART initiation		
Pre-pregnancy ART initiation		
HIV treatment (<2 or 2 years or more)	122	0
ART < 2 years		
ART ≥ 2 years or more		
GDM (yes)	337	9.4
Anaemia (yes)	372	0
Hypertension (yes)	372	0
**Birth outcomes**		
**Neonate sex**	372	0
Male		
Female		
Gestational age at time of enrolment (days)	372	0
Gestational age at delivery (weeks)	372	0
Mode of delivery	372	0
Vaginal		
Caesarean section		
**Anthropometry**		
Birth weight (kg)	369	0.8
Birth weight category[a]	369	0.8

Small-for-gestational age		
Appropriate-for-gestational age		
Large-for-gestational age		
Head circumference (cm)	369	0.8
Birth length (cm)	369	0.8
Apgar Score (>8); n(%)	369	0.8
**Placental parameters**		
Placental weight (g)	367	0.8
Placental efficiency	364	2.1
Placental length (cm)	371	0.3
Placental width (cm)	370	0.5
Placental roundness (cm)	368	1.1
Umbilical cord length (cm)	366	1.6
Umbilical cord diameter (cm)	364	2.2
**Feta growth parameters**		
Mean biparietal diameter size (cm)	372	0
Mean head circumference size (cm)	372	0
Mean abdominal circumference size (cm)	372	0
Mean femur length size (cm)	372	0
Mean biparietal diameter velocity (cm)	372	0
Mean head circumference velocity (cm)	372	0
Mean abdominal circumference velocity (cm)	372	0
Mean femur length size velocity (cm)	372	0

Table S2 Longitudinal fetal growth measurements at five time points during pregnancy, stratified by maternal HIV and ART status

	**Total**	**WNLWH (n=250, 67%)**	**WLWH (n=122, 33%)**	**P-value**
**Mean biparietal diameter (cm)**				
**14-18 weeks**	3.92 (0.42)	3.92 (0.42)	3.92 (0.42)	0.790
**19-23 weeks**	5.47 (0.42)	5.48 (0.42)	5.44 (0.42)	0.416
**24-28 weeks**	6.99 (0.41)	6.98 (0.42)	6.99 (0.41)	0.998
**29-33 weeks**	8.26 (0.41)	8.27 (0.41)	8.23 (0.42)	0.366
**34-38 weeks**	9.02 (0.44)	9.05 (0.42)	8.98 (0.48)	0.225
**Mean head circumference (cm)**				
**14-18 weeks**	13.80 (1.51)	13.78 (1.52)	13.82 (1.49)	0.100
**19-23 weeks**	19.43 (1.35)	19.44 (1.38)	19.39 (1.30)	0.612
**24-28 weeks**	24.74 (1.37)	24.74 (1.38)	24.74 (1.34)	0.717
**29-33 weeks**	29.10 (1.36)	29.21 (1.35)	28.88 (1.36)	0.040
**34-38 weeks**	31.75 (1.37)	31.84 (1.35)	31.54 (1.39)	0.101
**Mean abdominal circumference (cm)**				
**14-18 weeks**	11.45 (1.42)	11.45 (1.45)	11.46 (1.36)	0.948
**19-23 weeks**	16.65 (1.38)	16.67 (1.39)	16.62 (1.36)	0.761

**24-28 weeks**	21.9 (1.77)	21.83 (1.75)	22.03 (1.81)	0.643
**29-33 weeks**	27.12 (1.80)	27.12 (1.81)	27.11 (1.79)	0.972
**34-38 weeks**	31.79 (2.12)	31.67 (2.08)	32.08 (2.20)	0.141
**Mean femur length (cm)**				
**14-18 weeks**	2.35 (0.39)	2.33 (0.40)	2.37 (0.39)	0.393
**19-23 weeks**	3.69 (0.35)	3.69 (0.35)	3.69 (0.36)	0.970
**24-28 weeks**	4.88 (0.35)	4.87 (0.37)	4.91 (0.33)	0.403
**29-33 weeks**	6.00 (0.32)	5.99 (0.34)	6.02 (0.31)	0.505
**34-38 weeks**	6.78 (0.39)	6.77 (0.42)	6.81 (0.33)	0.456

Continuous data are presented as mean (SD). HIV, Human immunodeficiency virus. WLWH, Women living with HIV; WNLWH, Women not living with HIV.

Table S3 The longitudinal fetal growth measurements of male and female fetuses at five time points during pregnancy according to maternal HIV status

	**Males (n=197, 53)**			**Females (n=175, 47%)**		
**Mean biparietal diameter (cm)**	**Unexposed to HIV (n=138, 70%)**	**Exposed to HIV (n=59, 30%0**	**P-value**	**Unexposed to HIV (n=12, 64%)**	**Exposed to HIV (n=63, 36%)**	**P-value**
**14-18 weeks**	4.00 (0.44)	3.97 (0.45)	0.632	3.83 (0.39)	3.88 (0.38)	0.480
**19-23 weeks**	5.55 (0.43)	5.54 (0.41)	0.896	5.39 (0.39)	5.35 (0.41)	0.682
**24-28 weeks**	7.06 (0.41)	7.09 (0.41)	0.707	6.90 (0.41)	6.89 (0.38)	0.920
**29-33 weeks**	8.32 (0.40)	8.32 (0.39)	0.985	8.21 (0.40)	8.14 (0.40)	0.258
**34-38 weeks**	9.07 (0.44)	9.15 (0.48)	0.381	9.02 (0.41)	8.81 (0.43)	0.010
**Mean head circumference (cm)**						
**14-18 weeks**	14.03 (1.55)	14.02 (1.62)	0.947	13.48 (1.45)	13.63 (1.34)	0.517
**19-23 weeks**	19.64 (1.40)	19.63 (1.40)	0.972	19.21 (0.39)	19.16 (1.17)	0.653
**24-28 weeks**	25.05 (1.39)	25.17 (1.37)	0.837	24.38 (1.28)	24.36 (1.20)	0.786
**29-33 weeks**	29.37 (1.39)	29.31 (1.35)	0.802	29.02 (1.27)	28.47 (1.25)	0.008
**34-38 weeks**	31.98 (1.33)	32.07 (1.15)	0.728	31.68 (1.37)	31.04 (1.42)	0.016
**Mean abdominal circumference (cm)**						
**14-18 weeks**	11.76 (1.48)	11.66 (1.44)	0.671	11.07 (1.32)	11.26 (1.25)	0.373
**19-23 weeks**	16.87 (1.40)	16.78 (1.39)	0.677	16.41 (1.30)	16.48 (1.33)	0.896
**24-28 weeks**	22.09 (1.63)	22.39 (1.37)	0.474	21.52 (1.85)	21.70 (1.47)	0.654
**29-33 weeks**	27.17 (1.81)	27.22 (1.76)	0.889	27.06 (1.82)	27.02 (1.83)	0.884
**34-38 weeks**	31.73 (1.98(	32.51 (2.22)	0.046	31.59 (2.18)	31.67 (2.12)	0.845
**Mean femur length (cm)**						
**14-18 weeks**	2.39 (0.40)	2.39 (0.40)	0.971	2.27 (0.39)	2.36 (0.38)	0.224
**19-23 weeks**	3.71 (0.35)	3.70 (0.40)	0.940	3.67 (0.35)	3.69 (0.30)	0.703
**24-28 weeks**	4.91 (0.38_	4.94 (0.32)	0.792	4.83 (0.35)	4.89 (0.33)	0.247
**29-33 weeks**	6.00 (0.34)	6.03 (0.30)	0.566	5.99 (0.34)	6.01 (0.32)	0.709
**34-38 weeks**	6.76 (0.35)	6.87 (0.31)	0.081	6.78 (0.48)	6.75 (0.34)	0.468

Data are presented as mean (SD). ART, Antiretroviral therapy; HIV, Human immunodeficiency virus.

Table S4 The effect of HIV exposure on the size of fetal growth outcomes, with placental morphology variables as mediators in the combined sample

	**Effect of HIV exposure on:**	**Total effects (Path c)**		**Direct effects (Path c')**		**Indirect effects (Product of paths α and β, αβ)**	
		**Estimate (95% CI)**	***P*-value**	**Estimate (95% CI)**	***P*-value**	**Estimate (95% CI)**	***P*-value**
	**Head circumference (size)**						
Combined	Without placental weight	**-0.073 (-0.231-0.086)**	**0.368**				
	Via placental weight			-0.061 (-0.215-0.094)	0.443	-0.012 (-0.046-0.022)	0.489
	Without placental efficiency	**-0.088 (-0.245-0.070)**	**0.275**				
	Via placental efficiency			-0.089 (-0.246-0.067)	0.264	0.002 (-0.015-0.019)	0.854
	Without placental roundness	-0.073 (-0.231-0.086)	0.367				
	Via placental roundness			-0.073 (-0.232-0.085)	0.365	0.000 (-0.004-0.005)	0.887
	**Abdominal circumference (size)**						
Combined	Without placental weight	**0.069 (-0.126-0.265)**	**0.488**				
	Via placental weight			0.095 (-0.089-0.278)	0.312	-0.025 (-0.096-0.045)	0.485
	Without placental efficiency	**0.042 (-0.150-0.236)**	**0.666**				
	Via placental efficiency			0.042 (-0.151-0.235)	0.668	0.000 (-0.004-0.005)	0.866
	Without placental roundness	**0.098 (-0.099-0l.296)**	**0.328**				
	Via placental roundness			0.098 (-0.099-0.295)	0.329	0.000 (-0.004-0.005)	0.889
	**Biparietal diameter (size)**						
Combined	Without placental weight	**0.005 (-0.048-0.058)**	**0.846**				
	Via placental weight			0.009 (-0.043-0.061)	0.731	-0.004 (-0.015-0.007)	0.490
	Without placental efficiency	**0.001 (-0.052-0.054)**	**0.968**				
	Via placental efficiency			0.000 (-0.052-0.053)	0.986	0.001 (-0.006-0.007)	0.854
	Without placental roundness	**0.001 (-0.051-0.055)**	**0.957**				
	Via placental roundness			0.001 (-0.052-0.054)	0.964	0.000 (-0.003-0.003)	0.884
	**Femur length (size)**						
Combined	Without placental weight	**0.031 (-0.013-0.075)**	**0.163**				
	Via placental weight			0.034 (-0.09-0.078)	0.123	-0.003 (-0.011-0.005)	0.492
	Without placental efficiency	**0.024 (-0.018-0.067)**	**0.267**				
	Via placental efficiency			0.024 (-0.019-0.066)	0.272	0.000 (-0.003-0.004)	0.854
	Without placental roundness	**0.029 (-0.015-0.073)**	**0.199**				
	Via placental roundness			0.029 (-0.015-0.072)	0.197	-0.000 (-0.001-0.001)	0.888

Adjusted for age for the potential effects of fetal sex, gestational age at delivery, maternal age, GWG, parity and education. GWG, Gestational weight gain; HIV, Human immunodeficiency virus.

Table S5 The effect of HIV exposure on the velocity of fetal growth outcomes, with placental morphology variables as mediators in the combined sample

	**Effect of HIV exposure on:**	**Total effects (Path c)**		**Direct effects (Path c')**		**Indirect effects (Product of paths α and β, αβ)**	
		**Estimate (95% CI)**	***P*-value**	**Estimate (95% CI)**	***P*-value**	**Estimate (95% CI)**	***P*-value**
	**Head circumference (velocity)**						
Combined	Without placental weight	**-0.007 (-0.017-0.002)**	**0.139**				
	Via placental weight			-0.006 (-0.016-0.002)	0.172	-0.001 (-0.002-0.001)	0.490
	Without placental efficiency	**-0.008 (-0.018-0.001)**	**0.088**				
	Via placental efficiency			-0.008 (-0.018-0.001)	0.083	0.000 (-0.001-0.001)	0.854
	Without placental roundness	**-0.007 (-0.017-0.002)**	**0.137**				
	Via placental roundness			-0.007 (-0.017-0.002)	0.137	0.000 (-0.000-0.000)	0.938
	**Abdominal circumference (velocity)**						
Combined	Without placental weight	**0.007 (-0.008-0.022)**	**0.366**				
	Via placental weight			0.009 (-0.005-0.023)	0.212	-0.002 (-0.007-0.003)	0.485
	Without placental efficiency	**0.005 (-0.010-0.020)**	**0.510**				
	Via placental efficiency			0.005 (-0.010-0.020)	0.511	0.000 (-0.000-0.000)	0.956
	Without placental roundness	**0.010 (-0.005-0.025)**	**0.204**				
	Via placental roundness			0.010 (-0.005-0.025)	0.205	0.000 (-0.000-0.000)	0.923
	**Biparietal diameter (velocity)**						
Combined	Without placental weight	**-0.000 (-0.012-0.012)**	**0.951**				
	Via placental weight			0.000 (-0.011-0.012)	0.931	-0.000 (-0.003-0.002)	0.489
	Without placental efficiency	**-0.001 (-0.013-0.010)**	**0.811**				
	Via placental efficiency			-0.001 (-0.13-0.010)	0.790	0.000 (-0.001-0.001)	0.854
	Without placental roundness	**-0.001 (-0.014-0.010)**	**0.777**				
	Via placental roundness			-0.001 (-0.014-0.010)	0.774	0.000 (-0.000-0.000)	0.899
	**Femur length (velocity)**						
Combined	Without placental weight	**0.004 (-0.004-0.013)**	**0.313**				
	Via placental weight			0.004 (-0.003-0.013)	0.242	-0.000 (-0.002-0.001)	0.490
	Without placental efficiency	**0.003 (-0.005-0.011)**	**0.465**				
	Via placental efficiency			0.003 (-0.005-0.011)	0.469	0.000 (-0.000-0.000)	0.858
	Without placental roundness	**0.003 (-0.004-0.011)**	**0.420**				
	Via placental roundness			0.003 (-0.004-0.011)	0.415	-0.000 (-0.00-0.000)	0.884

Adjusted for age for the potential effects of fetal sex (only in the combined models), gestational age at delivery, maternal age, GWG, parity and education. GWG, Gestational weight gain; HIV, Human immunodeficiency virus.

Table S6 Path α estimates for the association between HIV status and placental morphology

Effect on HIV exposure on:	Estimate (95% CI)	P-value
**Male**		
Placental weight	0.235 (-30.755-31.225)	0.988
Placental efficiency	0.032 (-0.476-0.540)	0.902
Placental roundness	0.181 (-0.303-0.667)	0.463
**Female**		
Placental weight	-19.046 (-49.269-11.175)	0.217
Placental efficiency	0.120 (-0.236-0.477)	0.508
Placental roundness	-0.163 (-0.725-0.398)	0.569
**Combined**		
Placental weight	-7.785 (-29.541-13.971)	0.483
Placental efficiency	0.030 (-0.289-0.349)	0.854
Placental roundness	0.027 (-0.341-0.397)	0.883

Adjusted for age for the potential effects of fetal sex (only in the combined models), gestational age at delivery, maternal age, GWG, parity and education. GWG, Gestational weight gain; HIV, Human immunodeficiency virus.

Table S7 Path β estimates for the association between placental morphology and fetal growth outcomes (size and velocity)

	Head circumference (size)		Abdominal circumference (size)		Biparietal diameter (size)		Femur length (size)	
	**Estimate (95% CI)**	**P-value**	**Estimate (95% CI)**	**P-value**	**Estimate (95% CI)**	**P-value**	**Estimate (95% CI)**	**P-value**
**Male**								
Placental weight	0.001 (0.000-0.003)	0.000	0.003 (0.002-0.004)	0.000	0.000 (0.000-0.001)	0.001	0.000 (0.000-0.000)	0.005
Placental efficiency	0.051 (-0.012-0.115)	0.115	-0.000 (-0.073-0.071)	0.985	0.017 (-0.003-0.039)	0.104	0.010 (-0.006-0.027)	0.212
Placental roundness	0.037 (-0.030-0.106)	0.281	-0.008 (-0.086-0.069)	0.835	0.013 (-0.009-0.036)	0.249	0.003 (-0.014-0.021)	0.705
**Female**								
Placental weight	0.001 (-0.000-0.002)	0.060	0.002 (0.001-0.004)	0.000	0.000 (0.000-0.000)	0.052	0.000 (-0.000-0.000)	0.072
Placental efficiency	0.059 (-0.026-0.145)	0.172	0.035 (-0.085-0.157)	0.562	0.024 (-0.005-0.053)	0.106	0.019 (-0.006-0.044)	0.145
Placental roundness	-0.016 (-0.071-0.038)	0.551	0.026 (-0.051-0.104)	0.499	0.002 (-0.016-0.021)	0.818	-0.009 (-0.026-0.006)	0.236
**Combined**								
Placental weight	0.001 (0.000-0.002)	0.000	0.003 (0.002-0.004)	0.000	0.000 (0.000-0.000)	0.000	0.000 (0.000-0.000)	0.001
Placental efficiency	0.053 (0.003-0.103)	0.039	0.013 (-0.049-0.075)	0.676	0.020 (0.003-0.037)	0.020	0.012 (-0.001-0025)	0.082
Placental roundness	0.012 (-0.031-0.057)	0.567	0.012 (-0.042-0.067)	0.653	0.008 (-0.006-0.023)	0.257	-0.002 (-0.015-0.009)	0.639
	**Head circumference (velocity)**		**Abdominal circumference (velocity)**		**Biparietal diameter (velocity)**		**Femur length (velocity)**	
	**Estimate (95% CI)**	**P-value**	**Estimate (95% CI)**	**P-value**	**Estimate (95% CI)**	**P-value**	**Estimate (95% CI)**	**P-value**
**Male**								
Placental weight	0.000 (0.000-0.000)	0.000	0.000 (0.000-0.000)	0.000	0.000 (0.000-0.000)	0.000	0.000 (0.000-0.000)	0.000
Placental efficiency	0.002 (-0.001-0.006)	0192	-0.001 (-0.006-0.004)	0.721	0.003 (-0.001-0.008)	0.150	0.000 (-0.002-0.003)	0.830
Placental roundness	0.001 (-0.003-0.005)	0.601	-0.002 (-0.007-0.004)	0.610	0.001 (-0.004-0.006)	0.661	0.001 (-0.002-0.004)	0.610
**Female**								
Placental weight	0.000 (-0.000-0.000)	0.133	0.000 (-0.000-0.000)	0.000	0.000 (-0.000-0.000)	0.152	0.000 (-0.000-0.000)	0.115
Placental efficiency	0.004 (-0.001-0.009)	0.131	0.002 (-0.007-0.117)	0.667	0.008 (-0.001-0.008)	0.030	0.002 (-0.002-0.008)	0.297
Placental roundness	-0.001 (-0.004-0.002)	0.506	0.001 (-0.004-0.007)	0.633	0.000 (-0.004-0.004)	0.939	-0.003 (-0.006- -0.000)	0.038
**Combined**								
Placental weight	0.000 (0.000-0.000)	0.000	0.000 (0.000-0.000)	0.000	0.000 (0.000-0.000)	0.000	0.000 (0.000-0.000)	0.000
Placental efficiency	0.003 (-0.000-0.006)	0.051	0.003 (-0.000-0.006)	0.954	0.005 (0.001-0.009)	0.012	0.000 (-0.001-0.003)	0.463
Placental roundness	0.000 (-0.002-0.002)	0.926	0.000 (-0.004-0.005)	0.899	0.000 (-0.002-0.004)	0.626	-0.001 (-0.003-0.001)	0.318

Adjusted for age for the potential effects of fetal sex (only in the combined models), gestational age at delivery, maternal age, GWG, parity and education. GWG, Gestational weight gain; HIV, Human immunodeficiency virus.
